# Drosophila Rab39 Attenuates Lysosomal Degradation

**DOI:** 10.3390/ijms221910635

**Published:** 2021-09-30

**Authors:** Zsolt Lakatos, Péter Benkő, Gábor Juhász, Péter Lőrincz

**Affiliations:** 1 Department of Anatomy, Cell and Developmental Biology, Eötvös Loránd University, H-1117 Budapest, Hungary; lakzsolti@gmail.com (Z.L.); benkpeti@gmail.com (P.B.); 2 Department of Physiology, Semmelweis University, H-1094 Budapest, Hungary; 3 Biological Research Centre, Institute of Genetics, Hungarian Academy of Sciences, H-6726 Szeged, Hungary; 4 Premium Postdoctoral Research Program, Hungarian Academy of Sciences, H-1052 Budapest, Hungary

**Keywords:** Rab39, autophagy, endocytosis, lysosomes, Drosophila

## Abstract

Lysosomal degradation, the common destination of autophagy and endocytosis, is one of the most important elements of eukaryotic metabolism. The small GTPases Rab39A and B are potential new effectors of this pathway, as their malfunction is implicated in severe human diseases like cancer and neurodegeneration. In this study, the lysosomal regulatory role of the single Drosophila Rab39 ortholog was characterized, providing valuable insight into the potential cell biological mechanisms mediated by these proteins. Using a de novo CRISPR-generated *rab39* mutant, we found no failure in the early steps of endocytosis and autophagy. On the contrary, we found that Rab39 mutant nephrocytes internalize and degrade endocytic cargo at a higher rate compared to control cells. In addition, Rab39 mutant fat body cells contain small yet functional autolysosomes without lysosomal fusion defect. Our data identify Drosophila Rab39 as a negative regulator of lysosomal clearance during both endocytosis and autophagy.

## 1. Introduction

Lysosomal degradation pathways, autophagy, and endocytosis are essential for the proper homeostasis of all eukaryotic cells. Lysosomal malfunctions often lead to, or aggravate, severe human pathologies, such as Alzheimer’s disease or cancer [1]. Understanding the molecular mechanisms that govern lysosomal processes is of great clinical importance. Autophagic and endocytic degradation both depend on two key events: lysosomal fusion, and the subsequent clearance. Despite the numerous factors that are already identified, new, yet unknown proteins and mechanisms might emerge, providing new insights that can be utilized in tackling such diseases.

Ras-related in brain (Rab) proteins play a central role in vesicular transport and fusion events. Several Rab proteins are confirmed to regulate lysosomal pathways, e.g., Rab2 and Rab7 regulate lysosomal fusion and degradation, while Rab5 mediates early-to-late endosomal maturation [2,3,4,5]. Rab proteins convey effector responses in an active, GTP-bound state, thus, the factors regulating GTP- and GDP-binding, also have an excessive regulatory role [6]. Rab2 and Rab39 share a common ancestor and, therefore, are closely related, but in contrast to Rab2, the involvement of Rab39 in lysosome formation is poorly understood [7].

Rab39 was originally characterized as a trans-Golgi protein, where it regulates vesicle tethering, with possible pleiotropic effects in other vesicle trafficking pathways, such as endocytosis [8]. Mammalian organisms have two Rab39 paralogs, RAB39A and RAB39B, and the mutation of either leads to distinct pathologies. RAB39A depletion was shown to decrease the colonization rate of certain cancer stem cells, while augmenting lipopolysaccharide-induced autophagosome formation in macrophages [9,10,11,12]. RAB39A also regulates phagosome acidification and caspase-1 activity, and is also localized to lysosomal associated membrane protein 2 (LAMP2)-containing phagosomes [9,12,13]. RAB39B, on the other hand, is involved in the pathogenesis of an early-onset Parkinson-like disease, Waisman syndrome [14]. Both paralogs are implicated in proper neuritogenesis [15,16]. Interestingly, a GDP/GTP exchange factor (GEF) of RAB39B, C9ORF72 was found to regulate autophagy in neuronal cultures [17].

Gillingham et al. (2014) presented a comprehensive interactome of Drosophila Rab proteins, where they found strong interactions between Rab39 and endosomal maturation defective (Ema), as well as Unc-104, two proteins involved in endosomal maturation and lysosome motility, respectively [18]. Ema is an interacting partner of the homotypic fusion and vacuole protein sorting (HOPS) complex, and its depletion results in a lysosomal fusion defect similar to HOPS-deficient cells [19]. These results suggest that Rab39 may also have a role in lysosome formation similar to Rab2.

In this study, we characterize the role of the single Drosophila Rab39 ortholog regarding both autophagy and endocytosis using RNAi lines and a recently generated and published CRISPR/Cas9-generated *rab39* mutant allele [20]. Based on our results, we propose that Rab39 negatively regulates lysosomal degradation without altering endosomal or autophagic initiation and maturation.

## 2. Results

### 2.1. Rab39 Mutation Alters the Endolysosomal Distribution

**Figure 1:**
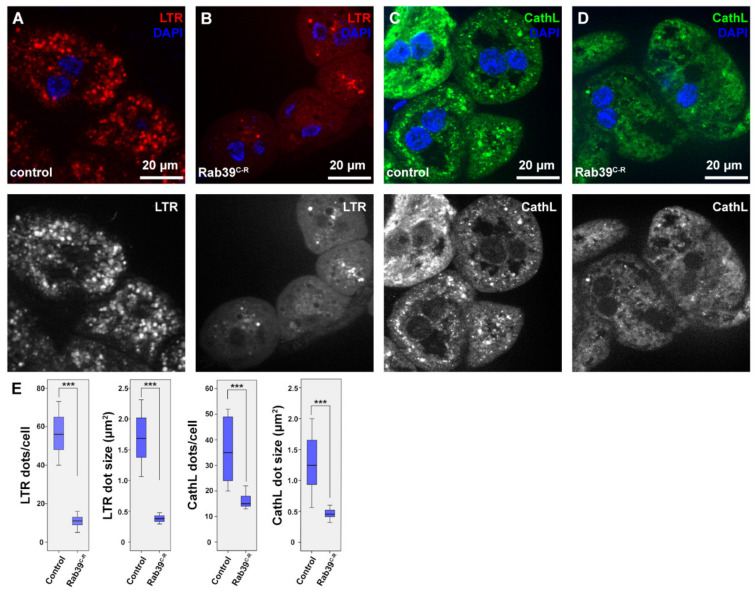
Endolysosomal distribution is altered in Rab39^C-R^ garland nephrocytes of L3 stage wandering larvae. (**A**,**B**) Acidic organelles were marked using LTR to depict endolysosomes in control (**A**) and Rab39^C-R^ (**B**) nephrocytes. Rab39 mutant nephrocytes contain significantly fewer and smaller LTR-positive vesicles. Please note that the smaller nuclei in B, when compared to A are only the result of natural deviation in nucleus size. (**C**,**D**) CathL was immunostained to show Golgi-derived vesicles, acidic and even non-acidic lysosomes in control (**C**) and Rab39 mutant (**D**) nephrocytes. CathL puncta were fewer and smaller in Rab39 mutant nephrocytes. (**A**–**D**) Respective red (**A**,**B**) and green (**C**–**D**) channels are shown in grayscale. (**E**) Quantifications of the LTR and CathL experiments, n = 10 cells. None of the samples showed Gaussian distribution. Medians are shown as horizontal black lines within the boxes. Bars show the upper and lower quartiles, and the whiskers plot the smallest and largest observations. *** *p* < 0.001. Heterozygous Rab39^C-R^ animals in A,C,E were used as controls.

We examined the morphology of the lysosomal compartment using two organs of *Drosophila melanogaster* larvae. During our studies, we used a CRISPR/Cas9 generated *rab39* mutant allele [20], in which the *rab39* coding sequence was replaced with an eyeless promoter-driven red fluorescent protein (RFP) gene. Thus, we named this allele *rab39^CRISPR-RFP^ (rab39^C-R^*). For the assessment of endolysosomes, we utilized the larval garland nephrocytes that continuously take up haemolymph via endocytosis [21]. First, we examined the acidic organelles using LysoTracker Red (LTR), as it detects acidic endolysosomes in nephrocytes. We found that LTR-positive puncta were smaller in number as well as in size in Rab39 mutant nephrocytes compared to the controls (Figure 1A,B,E).

In order to see whether the scarcity of LTR-positive vesicles is a result of defective acidification, we performed an immunostaining against the lysosomal hydrolase Cathepsin-L (CathL) that detects both acidic and non-acidic lysosomes. The size and number of CathL-positive puncta were also smaller in Rab39^C-R^ nephrocytes than in the control cells (Figure 1C–E). This suggests either an accelerated endolysosomal degradation or a reduced endocytic uptake or maturation, since lysosomal size increase is usually a sign of improper degradation [22,23].

### 2.2. Earlier Steps of Endocytosis Are Intact in Rab39 Mutant Nephrocytes

**Figure 2:**
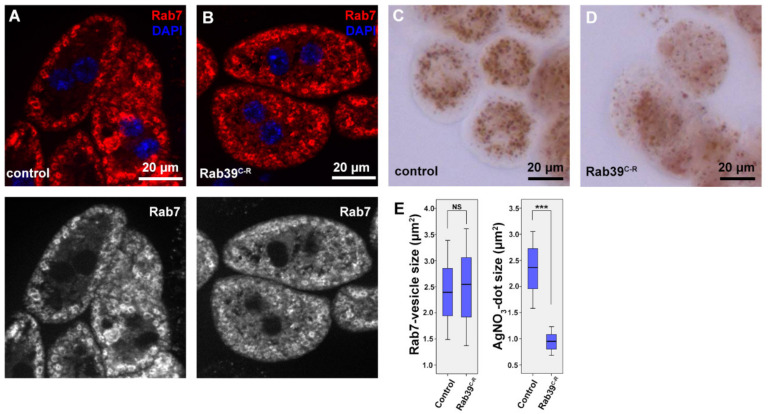
Endocytic uptake and maturation are unaffected in Rab39-depleted nephrocytes of L3 stage wandering larvae. (**A**,**B**) An immunostaining against late endosomal Rab7 was performed in control (**A**) and Rab39^C-R^ (**B**) cells. No visible alteration was detectable upon Rab39 deficiency, suggesting that endosomal maturation undergoes properly. The respective red channels are shown in grayscale. (**C**,**D**) Silver nitrate was taken up by control (**C**) or mutant (**D**) garland nephrocytes, where silver was detected in the form of a brown precipitate. Rab39 mutant nephrocytes could also incorporate silver nitrate, but in smaller vesicles, meaning that endocytic uptake is unaltered. (**E**) Quantification of the experiments depicted in (**A**,**D**), n = 10 cells. None of the samples showed Gaussian distribution. Medians are shown as horizontal black lines within the boxes. Bars show the upper and lower quartiles, and the whiskers plot the smallest and largest observations. NS: non-significant (*p* ≥ 0.05), *** *p* < 0.001. Heterozygous Rab39^C-R^ animals in A, C, E were used as control ones.

To assess what caused the observed lysosomal phenotype, we examined the previous steps of endocytosis in Rab39 mutant cells. Early endosomes were detected via immunostaining against Rab5, and we saw no difference between mutant and control cells (Figure S1A,B). An immunostaining against late endosomal Rab7 was also performed, which again displayed an intact late endosomal compartment in Rab39^C-R^ nephrocytes (Figure 2A,B,E). We next measured endocytic function of nephrocytes. In this experiment, the larvae were grown on silver nitrate-containing food, as this heavy metal is taken up from the haemolymph by nephrocytes [24]. Since heavy metals cannot be degraded in lysosomes, nephrocytes sequester them into terminal or storage lysosomes, or residual bodies, as shown in Figure 2C in the case of control cells. In Rab39 deprived cells, however, silver distribution is altered (Figure 2D,E). The silver containing vesicles show a scattered distribution, while their size is also decreased. These data verify that Rab39 mutant cells can initiate endocytosis, and up to the stage of late endosomes there is no detectable malfunction in this vesicular transport route. According to the silver uptake experiment, the later stages of lysosomal maturation might be altered.

### 2.3. Rab39 Depletion Accelerates Endosomal Maturation

**Figure 3:**
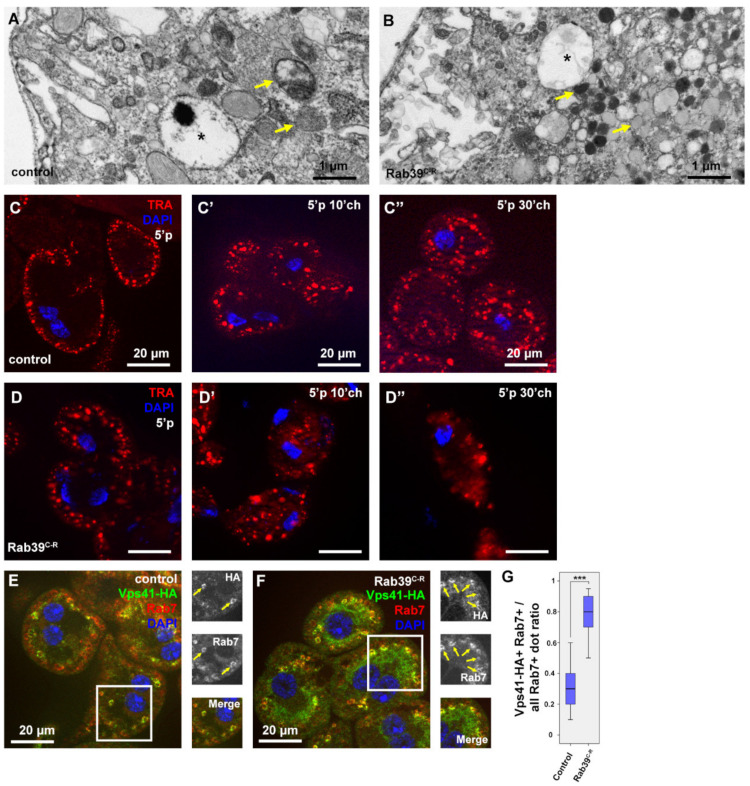
Endocytosis is accelerated in Rab39 depleted nephrocytes of L3 stage wandering larvae. (**A**,**B**) An ultrastructural analysis revealed normal late endosomal (marked with asterisks) size and distribution both in control (**A**) and Rab39 mutant (**B**) nephrocytes. In the mutant cells, however, only small, electron-dense lysosomes were present as opposed to control cells where lysosomes were larger. Two representative lysosomes are marked with arrows in both cases. (**C**,**D**) TRA was taken up by control (**C**) or Rab39 mutant (**D**) nephrocytes for 5 min, and it was chased 0 (**C**,**D**), 10 (**C**’,**D**’) or 30 min (**C**’’,**D**’’). In the mutant cells, TRA was internalized and degraded faster than in control cells. (**E**,**F**) The HA-tagged HOPS-subunit Vps41/Lt and late endosomal Rab7 were co-stained to detect their colocalization in control (**E**) and mutant cells (**F**). The colocalization increased upon Rab39 mutation. Boxed regions are enlarged in insets in (**E**,**F**) and colocalizing signals are marked with arrows. (**G**) Quantification of the experiments depicted in (**E**,**F**), n = 10 cells. None of the samples showed Gaussian distribution. Medians are shown as horizontal black lines within the boxes. Bars show the upper and lower quartiles, and the whiskers plot the smallest and largest observations. ***: *p* < 0.001. Heterozygous Rab39^C-R^ animals with (**E**,**G**) or without (**A**,**C**) the indicated transgenes were used as controls.

After having analyzed the nephrocyte morphology using fluorescent methods, their ultrastructure was also examined. We found a seemingly normal early and late endosomal compartment upon Rab39 depletion, as anticipated (Figure 3A,B). Nevertheless, the cytosol of the Rab39 mutant cells was abundant with small, electron-dense, lysosome-like vesicles (Figure 3B). These structures could be either Golgi-derived vesicles or some kind of lysosomes. Considering the small number of CathL-positive vesicles in mutant cells (Figure 1D), we suggest that only a small portion of the observed vesicles could be Golgi-derived vesicles (also known as primary lysosomes), the majority are more likely to be endolysosomes that have undergone degradation and are depleted of CathL.

To assess the degradation efficiency of Rab39 mutant cells, a fluorescent tracer, Texas Red-Avidin D (TRA), was added to the media in which the garland cells were incubated for 5 min (pulse). Afterward, the tracer was allowed to be internalized and degraded for an additional 0, 10, or 30 min (chase) by removing TRA from the media but keeping the cells within the media. In control cells after the 5 min pulse, TRA appeared in the outermost layer of the nephrocytes (Figure 3C). After the 10 min chase, TRA was observed closer to the nuclei and in larger vesicles that are presumably late endosomes and lysosomes (Figure 3C’) [4]. After 30 min of chase, TRA reached the perinuclear cytoplasm and most likely was sequestered by endolysosomes (Figure 3C’’) [19]. In Rab39 mutant cells, even after only the 5 min pulse, TRA was already present in larger vesicles that might be late endosomes (Figure 3D). It reached the perinuclear region after 10 min of chase and already started to be degraded, as the signal started to be vague and diffuse (3D’). After 30 min of chase, the TRA signal was considerably fainter in Rab39 mutant nephrocytes compared to the control cells, as most of the tracer was degraded and quenched by this time (3D’’). This suggests that TRA reaches lysosomes, and it is likely removed by accelerated lysosomal clearance in Rab39 mutants.

Increased lysosomal activity implies an elevated level of endosomal maturation, thus we assessed the colocalization between HOPS and late endosomal Rab7. In order to localize HOPS, a upstream activating sequence (UAS)-driven HA (hemagglutinin) epitope-tagged recombinant version of one of its subunits, Vps41/Light (Lt) was used [25]. We found increased colocalization between Vps41-HA and Rab7 upon Rab39 depletion in garland nephrocytes (Figure 3E,G), suggesting an accelerated late endosomal maturation that probably conveys late endosomes towards lysosomal fusion at a higher rate.

### 2.4. Rab39 Mutation Increases Autolysosomal Degradation in Fat Cells

**Figure 4:**
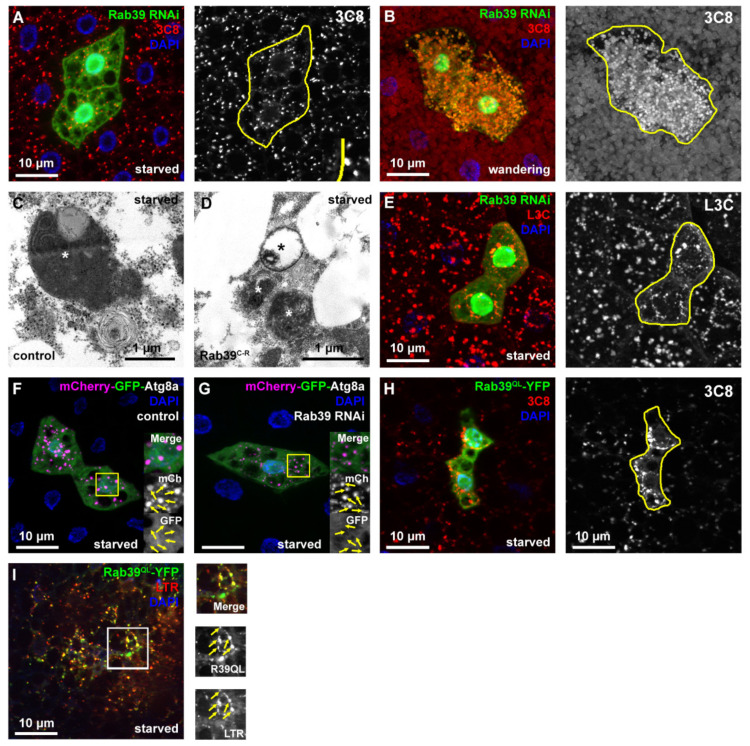
Rab39 accelerates autophagic degradation in fat cells. (**A**,**B**) 3xmCherry-Atg8a was expressed in all fat cells in starved (**A**) or wandering (**B**) L3 stage animals. In the GFP-positive cells, Rab39 was silenced with RNAi (marked with yellow line in the grayscale panels). In both cases, 3xmCherry-Atg8a dots were smaller in the GFP-positive cells compared to the adjacent control cells. (**A**) An inset shows the size difference of 3xmCherry-Atg8 dots. (**C**,**D**) Representative electron micrographs of autolysosomes (marked with asterisks) from control (**C**) and Rab39 mutant (**D**) starved fat cells of L3 stage wandering larvae. The mutant cells contain smaller yet degrading, functional autolysosomes (i.e., no intact organelles are visible within the autolysosome), and also no autophagosome accumulation is observed. (**E**) dLAMP-3xmCherry expressing fat body tissue from starved L3 stage larvae: Rab39 RNAi cells (marked with GFP) contain smaller dLAMP-3xmCherry dots than the neighboring control cells. (**F**,**G**) mCherry-GFP-Atg8a with (**G**) or without (**F**) Rab39 RNAi was expressed in several cells in fat tissues of L3 stage starved larvae. Magenta alone depicts autolysosomes (marked by arrows), as GFP is quenched in acidic environment. Please note that the difference in nuclei size is the result of rapid nuclei size change due to development and it does not affect autophagic flux. (**H**) Rab39^QL^-YFP was expressed in cell clones, while 3xmCherry-Atg8a reporter was expressed throughout the fat body tissue of L3 stage starved larvae. By expressing the constitutively active form of Rab39 in an ectopic manner, large mCherry-positive vesicles appeared. (**I**) Colocalization experiment in L3 stage starved larvae of Rab39^QL^-YFP and LTR shows a marginal overlap between the two signals, suggesting an autolysosomal Rab39 localization. Boxed regions are enlarged in (**F**,**G**,**I**) and colocalizing signals are marked by arrows. Heterozygous Rab39^C-R^ animals with (**F**) or without (**C**) the indicated transgenes were used as controls.

After having concluded that Rab39 might act as an inhibitory protein during endocytosis, we aimed to investigate autophagic degradation as well. We used our well-established methods in the fat body cells (hereafter referred to as fat cells) of third instar (L3) larvae, either in the early or wandering stages [26]. The former was starved in order to induce autophagy, while in case of the latter, developmental autophagy was already initiated, as wandering larvae break down their stored nutrients in a genetically programmed manner to produce sufficient energy during the pupal stage. In order to assess autophagy, first a 3xmCherry-tagged autophagy-related 8a (Atg8a) reporter (3C8) that labels all autophagic structures was used to detect alterations upon Rab39 depletion. To achieve the most precise results, a clonal system, where control and Rab39 RNAi cells were present in the same samples, was utilized [2]. Rab39 RNAi cells were marked with GFP, while the adjacent GFP-negative cells were control ones. Rab39-deficient cells of starved larvae contained considerably fainter and significantly fewer 3C8 vesicles, showing a clear lysosomal alteration (Figure 4A, Figure S2). When we turned to the same reporter in wandering larvae, 3C8 puncta were brighter, but nevertheless smaller in RNAi cells compared to the adjacent control ones (Figure 4B, Figure S2). Notably, the increased delivery of autophagic 3xmCherry-Atg8a toward lysosomes upon Raptor-mediated inhibition of TOR kinase complex 1 also produced smaller but brighter signal as a result of increased autophagic flux [27].

Our initial findings could also be explained by reduced lysosome biogenesis accompanied with autophagosome accumulation. We performed ultrastructural analysis in order to see whether the number of autophagosomes in starved early L3 stage larvae is increased. Our analysis revealed no change in autophagosome number. On the contrary, only small autolysosomes were observed, and within them cargo degradation progressed normally based on morphology, suggesting that there are no intact organelles present within (Figure 4C,D). Moreover, the distribution of autolysosomes was assessed using a dLAMP-3xmCherry (L3C) reporter construct in our clonal system in starved larvae. L3C puncta were smaller in Rab39 RNAi cells compared to the neighboring control cells (Figure 4E, Figure S2). Autophagic flux can be monitored using a tandem tagged Atg8a protein (mCherry-GFP-Atg8a), as GFP is pH-sensitive and thus gets quickly quenched in acidic environment, as opposed to the pH-stable mCherry [28,29]. We found no difference between Rab39 RNAi and control cells regarding autophagic flux, as GFP was quenched in both cases. Furthermore, as observed before, mCherry-positive vesicles are smaller in Rab39 RNAi cells (Figure 4F,G).

A UAS-driven, constitutively GTP-bound Rab39-YFP (Rab39^QL^-YFP) was utilized to examine the effects of Rab39 gain of function. Rab39^QL^-YFP enhanced the size of L3C puncta in starved fat cells in larvae, suggesting impaired lysosomal degradation (Figure 4H, Figure S2). RAB39A was shown to localize to LAMP2-vesicles in macrophages [9], thus we also wanted to verify the lysosomal localization of Drosophila Rab39. Rab39^QL^-YFP was expressed in fat cells, with acidic lysosomes marked by LTR. The two signals showed partial colocalization, demonstrating that a subset of lysosomes contains Rab39 on their limiting membranes (Figure 4I).

## 3. Discussion

Factors governing lysosomal degradation have long been investigated, as their malfunctions often lead to numerous kinds of pathologies. With the most common ones being neurodegenerative and cancerous diseases, the clinical relevance of characterizing these proteins is paramount. With each discovered regulatory element, a potential new therapeutic factor emerges. In the past years, several proteins residing primarily in the Golgi or Golgi-derived vesicles were claimed to play a role in lysosomal degradation and some of these proteins were characterized by our group, such as Rab2 which is closely related to Rab39 [4,30].

Rab39 also localizes predominantly to the trans-Golgi, nevertheless, it was claimed to be involved in a variety of cellular mechanisms, e.g., endocytosis, neuritogenesis, oxidative stress response, cannabinoid receptor signaling, Drosophila embryogenesis, Notch signaling, lipid transport, or microtubular organization [11,31,32,33,34].

In this work, we propose a role of Drosophila Rab39 as a negative regulator of lysosomal degradation during both endocytosis and autophagy. This finding is in line with an article that shows that in RAB39A-depleted macrophages, lipopolysaccharide-induced autophagy is augmented, while RAB39A localizes to LAMP2-vesicles [9]. The mechanism by which Rab39 proteins regulate autophagy remains to be elucidated. A possible pathway of this mechanism could be the regulation of the mechanistic target of rapamycin (mTOR) or transcription factor EB (TFEB), as they both reside on lysosomes and modulate lysosomal biogenesis and clearance [35,36,37]. Another possible mechanism could be the regulation of cellular localization of lysosomes, as Rab39 interacts with the kinesin-like protein KIF1A (Drosophila Unc-104). KIF1A was shown to transport Atg9 to presynaptic vesicles and thus plays a central role in neurodevelopment and neural function maintenance [18,38]. This interaction could prove as possible explanation for the observed neurodegeneration upon RAB39B mutation. It was shown both in Drosophila and humans that KIF1A mutation causes neuropathies, as it is also involved in synaptic vesicle transport [39,40]. Furthermore, both Unc-104 and Rab39 interact with Pruning defect 1 (Prd1) that participates in the endolysosomal degradation of excessive axons and dendrites in association with Unc-104 [41]. Rab39 was found to interact with the dynein-adaptor Bicaudal D (BicD), which was also shown to bind Rab2, a Golgi-derived protein involved in lysosomal degradation [4,18]. Furthermore, according to our findings, the HOPS subunit Vps41 was enriched on late endosomes in Rab39-depleted cells. A possible explanation could be that a clear interaction was found between Rab39 and Ema, an effector of the HOPS complex [18]. Since Ema was found to play a crucial role in both autophagy and endocytosis [19,42], a connection between Ema and Rab39 seems suitable in the regulation of both processes.

In conclusion, the interaction network of Rab39 could underlie various mechanisms by which Rab39 fine tunes lysosomal clearance. In our Drosophila model cells, a clear enhanced lysosomal degradation phenotype was observed in Rab39 loss-of-function cells. On the other hand, it remains to be explored how Rab39 could serve as a hub in association with Unc-104, Prd1, Ema, and BicD.

## 4. Materials and Methods

### 4.1. Fly Work

Flies were raised at 25 °C on regular cornmeal–agar–yeast medium. To acquire starving fat cells, 95 h old larvae were starved in 20% sucrose solution for 4 h at room temperature (RT). For obtaining wandering fat body tissue, where developmental autophagy occurs, and for the nephrocyte experiments, wandering L3 instar larvae were dissected. The Rab39^C-R^ stock was a gift of Peter Robin Hiesinger (Freie Universität Berlin, Germany). Rab39 RNAi (FlyBase ID: FBst0053995) and Rab39^QL^-YFP (FlyBase ID: FBst0009823) were obtained from the Bloomington Drosophila Stock Center (BDSC), Bloomington, IN, USA. We generated Gal4-expressing fat cell clones using the following stocks: hs-Flp; UAS-DCR2, Act > CD2 > Gal4; UAS-GFP, or hs-Flp; dLAMP-3x-mCherry, UAS-GFP; Act > CD2 > Gal4, UAS-DCR2, or hs-Flp; 3x-mCherry-Atg8a, UAS-GFP; Act > CD2 > Gal4, UAS-DCR2 [2,43]. UAS-Vps41-9xHA was generated in our lab [30]. Autophagic flux was assessed using the tandem GFP-mCherry-Atg8a reporter as described earlier [27,44]. The prospero-Gal4 stock for expression in nephrocytes was a gift of Bruce Edgar (ZMBH, Heidelberg, Germany).

The generation of fat cell clones is bosed on the random cellular expression of the heat shock (hs) promoter driven Flip recombinase (Flp). In a subset of cells, Flp eliminates the CD2 casette from between the actin (Act) promoter and the Gal4 coding sequence, thus enabling Gal4 transcription. Upon Gal4 expression, genes driven by the UAS promoter are also expressed in the same cell (i.e., GFP that marks the cell, and the RNAi/overexpression construct).

### 4.2. Immunohistochemistry

For immunostaining, larvae were bisected in phosphate buffered saline (PBS) and fixed with 4% formaldehyde in PBS for 50 min at (RT). Samples were rinsed and extensively washed (3 × 10 min at RT) and permeabilized in PBS with 0.1% Triton X-100 (PBTX) for 10 min at RT, followed by incubation in blocking solution (5.0% FCS (DE14-802F, Lonza, Belgium) in PBTX, 30 min at RT). Samples were then incubated in blocking solution containing primary antibodies and sodium azide (0.01%) overnight at 4 °C. Next, samples were rinsed 3 times and washed with PBTX with extra 4% NaCl for 15 min at RT, then washed with PBTX for 3 × 15 min in PBTX at RT, then incubated in blocking solution for 30 min at RT, followed by incubation with secondary antibodies in blocking solution for 3 h at RT. Washing steps were repeated, while nuclei were stained using 4′,6-diamidino-2-phenylindole (DAPI) added to the NaCl-containing PBTX, and samples were mounted in Vectashield (Vector Laboratories, Burlingame, CA, USA, H-1000). The primary antibodies were: monoclonal mouse anti-Rab7 (1:10, Developmental Studies Hybridoma Bank/DSHB, Iowa City, IA, USA) [45], polyclonal rabbit anti-CathL (1:100; ab58991, Abcam, Cambridge, UK), polyclonal rabbit anti-HA 1:100 (Merck, Darmstadt, Germany, H6908), polyclonal rabbit anti-Rbsn-5 (1:1000) [46]. The secondary antibodies were: Alexa Fluor 568 goat anti-mouse, Alexa Fluor 488 goat anti-rabbit (all 1:1000; Invitrogen, Carlsbad, CA, USA).

### 4.3. LysoTracker Staining and TRA Uptake Assay

For TexasRed-Avidin uptake assay, L3 wandering larval proventriculi with garland nephrocytes were prepared in cold Shields and Sang M3 insect medium (hereafter referred to as M3, Merck, Darmstadt, Germany, S8398) and incubated in 0.1 mg/mL TexasRed-Avidin D (Vector Laboratories, A-2006) containing M3 for 5 min at RT, rinsed 3 times, then incubated in M3 for 0, 10 or 30 min, then fixed with 4% formaldehyde in PBS for 50 min at RT. Samples were washed 3 × 10 min in PBS, stained with DAPI and mounted as described before. For LysoTracker staining, fat tissues of starved larvae were bisected in cold PBS and incubated in LysoTracker Red DND-99 (1:1000 in PBS; Thermo Fisher Scientific, L7528) for 1 min at RT. Samples were then rinsed 3 times, mounted in 80% glycerol in PBS containing DAPI, and photographed immediately.

### 4.4. Fluorescent Imaging

Fluorescent images were obtained at RT with an AxioImager.M2 microscope (Carl Zeiss, Oberkochen, Germany) with an ApoTome2 grid confocal unit (Carl Zeiss) using EC Plan-Neofluar 40×/0.75-NA Air (Carl Zeiss) or Plan-Apochromat 40×/0.95-NA Air (Carl Zeiss) objectives for fat cells, and Plan-Apochromat 63×/1.40-NA Oil (Carl Zeiss) objective for nephrocytes, an Orca Flash 4.0 LT sCMOS camera (Hamamatsu Photonics, Hamamatsu, Japan), and Zeiss Efficient Navigation 2 software (Carl Zeiss). Immersol 518F (Carl Zeiss) immersion oil was used for the 63x objective. Images from 8 consecutive focal planes (section thickness: 0.25 µm for nephrocytes and 0.35 µm for fat cells) were projected onto one single image, except for the colocalization assays, where we aimed to exclude any false positive colocalization, thus assessing only one focal plane. Images were processed in Zeiss Efficient Navigation 2 (Carl Zeiss) and Photoshop CS4 or CS6 (Adobe, San Jose, CA, USA) to present final figures.

### 4.5. Electron Microscopic Analysis

Fat bodies or nephrocytes were fixed in a solution containing 3.2% paraformaldehyde, 0.5% or 1% glutaraldehyde for nephrocytes or fat cells, respectively, 1% sucrose, and also 0.028% CaCl_2_ in 0.1 N sodium cacodylate, pH 7.4, overnight at 4 °C. Samples were immobilized within 1% agar in distilled water (DW). Samples were postfixed and contrasted in 0.5% OsO_4_ for 1 h and in half-saturated aqueous uranyl acetate for 30 min at RT, dehydrated in a graded series (50%, 70%, 96% and 100%) of ethanol, and embedded in Durcupan (Fluka, Buchs, Switzerland) according to the manufacturer’s protocols. Then, 70 nm sections were stained in Reynolds’ lead citrate and viewed with a JEM-1011 transmission electron microscope (Jeol, Tokyo, Japan) with a Morada digital camera (Olympus, Tokyo, Japan) using iTEM software (Olympus). Images were processed in Photoshop CS4 (Adobe) to present final figures.

### 4.6. Statistics

We quantified our unmodified, single focal plane images using ImageJ software (NIH, Bethesda, MD, USA). n = ten cells of each genotype were quantified. For measurement, cells were randomly selected, and the areas were measured manually. In the mosaic animal experiments, a randomly generated RNAi cell clone and a neighboring control cell was chosen for quantification. The normality test for each data sets was performed using SPSS17 (IBM, Armonk, NY, USA), and when the distribution of at least one of the compared datasets was non-Gaussian, the Mann–Whitney U-test was performed. When each dataset showed normal distribution, T-test with independent samples was performed.

## Data Availability

Not applicable.

## Supplementary Materials

The following are available online at https://www.mdpi.com/article/10.3390/ijms221910635/s1, Supplementary Figure S1: Early endosomal distribution is unaltered in Rab39 mutant nephrocytes. (A-B) Early endosomal Rab5 was marked by immunostaining both in control (A) and Rab39^C-R^ (B) garland nephrocytes with no difference in vesicle size or distribution. Supplementary Figure S2. Statistical analysis of experiments depicted in Figure 4: From left to right: number of 3xmCherry-Atg8a dots per cell in starved larvae (non-Gaussian), size of 3xmCherry-Atg8a dots in starved larvae (non-Gaussian), number of 3xmCherry-Atg8a dots per cell in wandering larvae (non-Gaussian), size of 3xmCherry-Atg8a dots in wandering larvae (non-Gaussian), size of dLAMP-3xmCherry dots in starved larvae (Gaussian), size of 3xmCherry-Atg8a dots in starved larvae (Gaussian). In all cases n = 10 cells. Medians are shown as horizontal black lines within the boxes. Bars show the upper and lower quartiles, and the whiskers plot the smallest and largest observations. ** *p* < 0.01 *** *p* < 0.001.

## References

[1] Ballabio A., Bonifacino J.S. (2020). Lysosomes as dynamic regulators of cell and organismal homeostasis. Nat. Rev. Mol. Cell Biol..

[2] Hegedűs K., Takáts S., Boda A., Jipa A., Nagy P., Varga K., Kovács A.L., Juhász G. (2016). The Ccz1-Mon1-Rab7 module and Rab5 control distinct steps of autophagy. Mol. Biol. Cell.

[3] Fujita N., Huang W., Lin T.-h., Groulx J.-F., Jean S., Nguyen J., Kuchitsu Y., Koyama-Honda I., Mizushima N., Fukuda M. (2017). Genetic screen in Drosophila muscle identifies autophagy-mediated T-tubule remodeling and a Rab2 role in autophagy. Elife.

[4] Lőrincz P., Tóth S., Benkő P., Lakatos Z., Boda A., Glatz G., Zobel M., Bisi S., Hegedűs K., Takáts S. (2017). Rab2 promotes autophagic and endocytic lysosomal degradation. J. Cell Biol..

[5] Bucci C., Parton R.G., Mather I.H., Stunnenberg H., Simons K., Hoflack B., Zerial M. (1992). The small GTPase rab5 functions as a regulatory factor in the early endocytic pathway. Cell.

[6] Barr F., Lambright D.G. (2010). Rab gefs and gaps. Curr. Opin. Cell Biol..

[7] Klöpper T.H., Kienle N., Fasshauer D., Munro S. (2012). Untangling the evolution of Rab G proteins: Implications of a comprehensive genomic analysis. BMC Biol..

[8] Chen T., Han Y., Yang M., Zhang W., Li N., Wan T., Guo J., Cao X. (2003). Rab39, a novel Golgi-associated Rab GTPase from human dendritic cells involved in cellular endocytosis. Biochem. Biophys. Res. Commun..

[9] Seto S., Sugaya K., Tsujimura K., Nagata T., Horii T., Koide Y. (2013). Rab39a interacts with phosphatidylinositol 3-kinase and negatively regulates autophagy induced by lipopolysaccharide stimulation in macrophages. PLoS ONE.

[10] Chano T., Avnet S. (2018). RAB39A: A Rab small GTPase with a prominent role in cancer stemness. J. Biochem..

[11] Chano T., Kita H., Avnet S., Lemma S., Baldini N. (2018). Prominent role of RAB39A-RXRB axis in cancer development and stemness. Oncotarget.

[12] Seto S., Tsujimura K., Koide Y. (2011). Rab GTPases regulating phagosome maturation are differentially recruited to mycobacterial phagosomes. Traffic.

[13] Becker C.E., Creagh E.M., O’Neill L.A. (2009). Rab39a binds caspase-1 and is required for caspase-1-dependent interleukin-1β secretion. J. Biol. Chem..

[14] Wilson G.R., Sim J.C., McLean C., Giannandrea M., Galea C.A., Riseley J.R., Stephenson S.E., Fitzpatrick E., Haas S.A., Pope K. (2014). Mutations in RAB39B cause X-linked intellectual disability and early-onset Parkinson disease with α-synuclein pathology. Am. J. Hum. Genet..

[15] Giannandrea M., Bianchi V., Mignogna M.L., Sirri A., Carrabino S., D′Elia E., Vecellio M., Russo S., Cogliati F., Larizza L. (2010). Mutations in the small GTPase gene RAB39B are responsible for X-linked mental retardation associated with autism, epilepsy, and macrocephaly. Am. J. Hum. Genet..

[16] Mori Y., Matsui T., Omote D., Fukuda M. (2013). Small GTPase Rab39A interacts with UACA and regulates the retinoic acid-induced neurite morphology of Neuro2A cells. Biochem. Biophys. Res. Commun..

[17] Corbier C., Sellier C. (2017). C9ORF72 is a GDP/GTP exchange factor for Rab8 and Rab39 and regulates autophagy. Small GTPases.

[18] Gillingham A.K., Sinka R., Torres I.L., Lilley K.S., Munro S. (2014). Toward a comprehensive map of the effectors of rab GTPases. Dev. Cell.

[19] Kim S., Wairkar Y.P., Daniels R.W., DiAntonio A. (2010). The novel endosomal membrane protein Ema interacts with the class C Vps–HOPS complex to promote endosomal maturation. J. Cell Biol..

[20] Kohrs F.E., Daumann I.-M., Pavlovic B., Jin E.J., Kiral F.R., Lin S.-C., Port F., Wolfenberg H., Mathejczyk T.F., Linneweber G.A. (2021). Systematic functional analysis of rab GTPases reveals limits of neuronal robustness to environmental challenges in flies. Elife.

[21] Weavers H., Prieto-Sánchez S., Grawe F., Garcia-López A., Artero R., Wilsch-Braeuninger M., Ruiz-Gómez M., Skaer H., Denholm B. (2009). The insect nephrocyte is a podocyte-like cell with a filtration slit diaphragm. Nature.

[22] Mauvezin C., Nagy P., Juhász G., Neufeld T.P. (2015). Autophagosome–lysosome fusion is independent of V-ATPase-mediated acidification. Nat. Commun..

[23] Maruzs T., Lőrincz P., Szatmári Z., Széplaki S., Sándor Z., Lakatos Z., Puska G., Juhász G., Sass M. (2015). Retromer ensures the degradation of autophagic cargo by maintaining lysosome function in Drosophila. Traffic.

[24] Lőrincz P., Lakatos Z., Varga Á., Maruzs T., Simon-Vecsei Z., Darula Z., Benkő P., Csordás G., Lippai M., Andó I. (2016). MiniCORVET is a Vps8-containing early endosomal tether in Drosophila. Elife.

[25] Lőrincz P., Kenéz L.A., Tóth S., Kiss V., Varga Á., Csizmadia T., Simon-Vecsei Z., Juhász G. (2019). Vps8 overexpression inhibits HOPS-dependent trafficking routes by outcompeting Vps41/Lt. Elife.

[26] Lőrincz P., Mauvezin C., Juhász G. (2017). Exploring autophagy in Drosophila. Cells.

[27] Nagy P., Varga Á., Kovács A.L., Takáts S., Juhász G. (2015). How and why to study autophagy in Drosophila: It’s more than just a garbage chute. Methods.

[28] Kimura S., Noda T., Yoshimori T. (2007). Dissection of the autophagosome maturation process by a novel reporter protein, tandem fluorescent-tagged LC3. Autophagy.

[29] Doherty G.P., Bailey K., Lewis P.J. (2010). Stage-specific fluorescence intensity of GFP and mCherry during sporulation in Bacillus subtilis. BMC Res. Notes.

[30] Lakatos Z., Lőrincz P., Szabó Z., Benkő P., Kenéz L.A., Csizmadia T., Juhász G. (2019). Sec20 is required for autophagic and endocytic degradation independent of Golgi-ER retrograde transport. Cells.

[31] Millage M.R. (2020). Rab39 and Klp98a Are Required for Furrow Formation During Early Drosophila Embryogenesis. Ph.D. Thesis.

[32] Caviglia S., Brankatschk M., Fischer E.J., Eaton S., Luschnig S. (2016). Staccato/Unc-13-4 controls secretory lysosome-mediated lumen fusion during epithelial tube anastomosis. Nat. Cell Biol..

[33] Best B.T., Leptin M. (2020). Multiple requirements for Rab GTPases in the development of Drosophila tracheal dorsal branches and terminal cells. G3 Genes Genomes Genet..

[34] Gambarte Tudela J., Buonfigli J., Luján A., Alonso Bivou M., Cebrián I., Capmany A., Damiani M.T. (2019). Rab39a and Rab39b display different intracellular distribution and function in sphingolipids and phospholipids transport. Int. J. Mol. Sci..

[35] Settembre C., Zoncu R., Medina D.L., Vetrini F., Erdin S., Erdin S., Huynh T., Ferron M., Karsenty G., Vellard M.C. (2012). A lysosome-to-nucleus signalling mechanism senses and regulates the lysosome via mTOR and TFEB. EMBO J..

[36] Roczniak-Ferguson A., Petit C.S., Froehlich F., Qian S., Ky J., Angarola B., Walther T.C., Ferguson S.M. (2012). The transcription factor TFEB links mTORC1 signaling to transcriptional control of lysosome homeostasis. Sci. Signal..

[37] Averous J., Lambert-Langlais S., Carraro V., Gourbeyre O., Parry L., B′Chir W., Muranishi Y., Jousse C., Bruhat A., Maurin A.-C. (2014). Requirement for lysosomal localization of mTOR for its activation differs between leucine and other amino acids. Cell. Signal..

[38] Stavoe A.K., Hill S.E., Hall D.H., Colón-Ramos D.A. (2016). KIF1A/UNC-104 transports ATG-9 to regulate neurodevelopment and autophagy at synapses. Dev. Cell.

[39] Rivière J.-B., Ramalingam S., Lavastre V., Shekarabi M., Holbert S., Lafontaine J., Srour M., Merner N., Rochefort D., Hince P. (2011). KIF1A, an axonal transporter of synaptic vesicles, is mutated in hereditary sensory and autonomic neuropathy type 2. Am. J. Hum. Genet..

[40] Zhang Y.V., Hannan S.B., Kern J.V., Stanchev D.T., Koç B., Jahn T.R., Rasse T.M. (2017). The KIF1A homolog Unc-104 is important for spontaneous release, postsynaptic density maturation and perisynaptic scaffold organization. Sci. Rep..

[41] Zong W., Wang Y., Tang Q., Zhang H., Yu F. (2018). Prd1 associates with the clathrin adaptor α-Adaptin and the kinesin-3 Imac/Unc-104 to govern dendrite pruning in Drosophila. PLoS Biol..

[42] Kim S., Naylor S.A., DiAntonio A. (2012). Drosophila Golgi membrane protein Ema promotes autophagosomal growth and function. Proc. Natl. Acad. Sci. USA.

[43] Korolchuk V.I., Saiki S., Lichtenberg M., Siddiqi F.H., Roberts E.A., Imarisio S., Jahreiss L., Sarkar S., Futter M., Menzies F.M. (2011). Lysosomal positioning coordinates cellular nutrient responses. Nat. Cell Biol..

[44] Mauvezin C., Ayala C., Braden C.R., Kim J., Neufeld T.P. (2014). Assays to monitor autophagy in Drosophila. Methods.

[45] Riedel F., Gillingham A.K., Rosa-Ferreira C., Galindo A., Munro S. (2016). An antibody toolkit for the study of membrane traffic in Drosophila melanogaster. Biol. Open.

[46] Tanaka T., Nakamura A. (2008). The endocytic pathway acts downstream of Oskar in Drosophila germ plasm assembly. Development.

