# Supplementary material for: Drosophila Rab39 Attenuates Lysosomal Degradation
Source: Int J Mol Sci. 2021 Sep 30;22(19):10635. doi: 10.3390/ijms221910635 (PMC8508792; doi:10.3390/ijms221910635)
Supplement: Supplementary file 1 [file ijms-22-10635-s001.zip › Supplementary figure legends.pdf]

Supplementary figure legends

Supplementary figure S1. Early endosomal distribution is unaltered in Rab39 mutant nephrocytes. (A-B) Early endosomal Rab5 was marked by immunostaining both in control (A) and Rab39C-R (B) garland nephrocytes with no difference in vesicle size or distribution.

Supplementary figure S2. Statistical analysis of experiments depicted in Fig. 4. From left to right: number of 3xmCherry-Atg8a dots per cell in starved larvae (non-Gaussian), size of 3xmCherry-Atg8a dots in starved larvae (non-Gaussian), number of 3xmCherry-Atg8a dots per cell in wandering larvae (non-Gaussian), size of 3xmCherry-Atg8a dots in wandering larvae (non-Gaussian), size of dLAMP-3xmCherry dots in starved larvae (Gaussian), size of 3xmCherry-Atg8a dots in starved larvae (Gaussian). In all cases n = 10 cells. Medians are shown as horizontal black lines within the boxes. Bars show the upper and lower quartiles, and the whiskers plot the smallest and largest observations. ** $p<0.01$ *** $p<0.001$.
