# Supplementary figures and images for: Drosophila Rab39 Attenuates Lysosomal Degradation
Source: Int J Mol Sci. 2021 Sep 30;22(19):10635. doi: 10.3390/ijms221910635 (PMC8508792; doi:10.3390/ijms221910635)

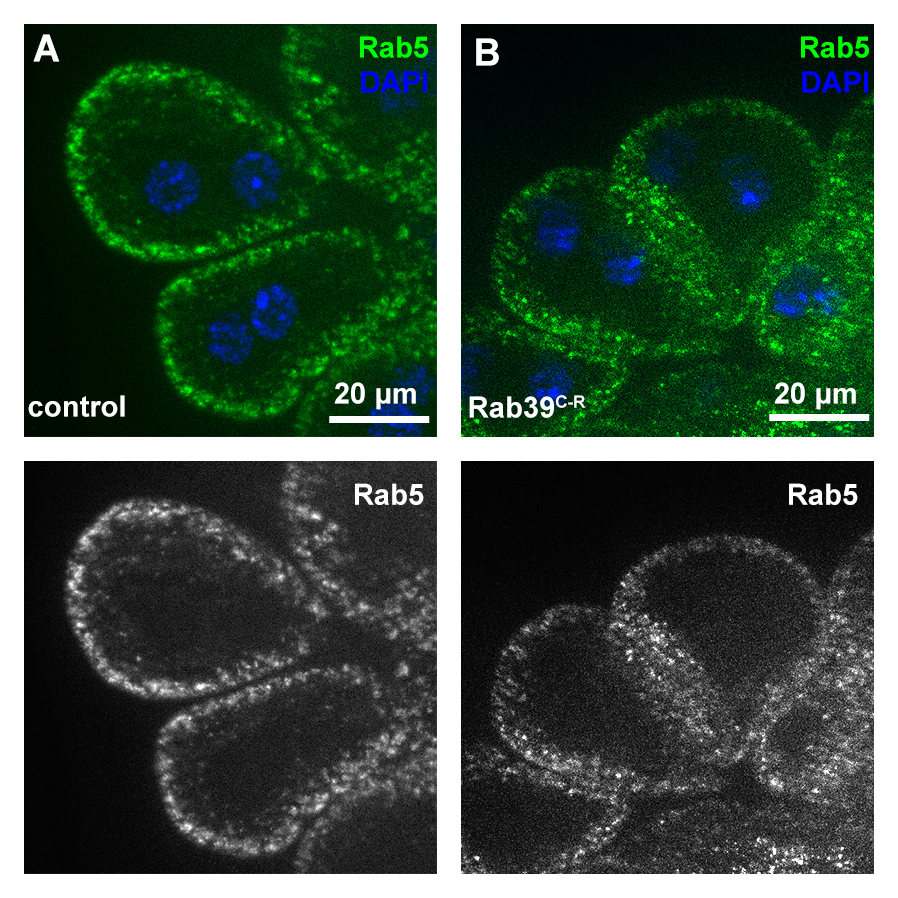

Supplement: Supplementary file 1 [file ijms-22-10635-s001.zip › fig_s1.tif]

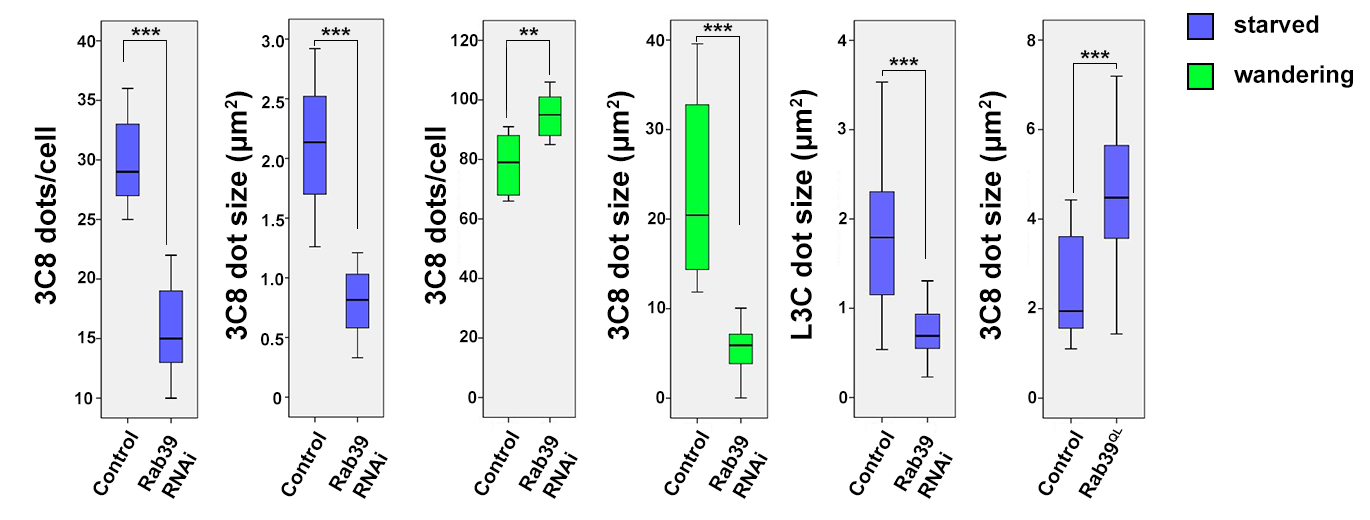

Supplement: Supplementary file 1 [file ijms-22-10635-s001.zip › fig_s2.tif]
